# 

**Published:** 1890-05

**Authors:** 


MAY, 1890.

(108)

### THE AMERICAN JOURNAL OF DENTAL SCIENCE.

PUBLISHED MONTHLY.

$2.50 PER ANNUM.

EDITED BY

F. J. S. GORGAS, M. D., D. D. S.

Vol. 24. THIRD SERIES. No. 1.

SURGEON GENL'S OFFICE LIBRARY

BALTIMORE:
SNOWDEN & COWMAN, 9 W. FAYETTE STREET.

LONDON:
TRUBNER & CO., 60 PATERNOSTER ROW.

PAYABLE IN ADVANCE.

